# Recent Trends in Compressive Raman Spectroscopy Using DMD-Based Binary Detection

**DOI:** 10.3390/jimaging5010001

**Published:** 2018-12-21

**Authors:** Derya Cebeci, Bharat R. Mankani, Dor Ben-Amotz

**Affiliations:** 1 PortMera Corp., Stony Brook, NY 11790, USA; 2 MarqMetrix Inc., Seattle, WA 98103, USA; 3 Department of Chemistry, Purdue University, West Lafayette, IN 47907, USA

**Keywords:** Raman spectroscopy, chemical imaging, compressive detection, spatial light modulators (SLM), digital micromirror device (DMD), digital light processor (DLP), optimal binary filters, Chemometrics, multivariate data analysis

## Abstract

The collection of high-dimensional hyperspectral data is often the slowest step in the process of hyperspectral Raman imaging. With the conventional array-based Raman spectroscopy acquiring of chemical images could take hours to even days. To increase the Raman collection speeds, a number of compressive detection (CD) strategies, which simultaneously sense and compress the spectral signal, have recently been demonstrated. As opposed to conventional hyperspectral imaging, where full spectra are measured prior to post-processing and imaging CD increases the speed of data collection by making measurements in a low-dimensional space containing only the information of interest, thus enabling real-time imaging. The use of single channel detectors gives the key advantage to CD strategy using optical filter functions to obtain component intensities. In other words, the filter functions are simply the optimized patterns of wavelength combinations characteristic of component in the sample, and the intensity transmitted through each filter represents a direct measure of the associated score values. Essentially, compressive hyperspectral images consist of ‘score’ pixels (instead of ‘spectral’ pixels). This paper presents an overview of recent advances in compressive Raman detection designs and performance validations using a DMD based binary detection strategy.

## 1. Introduction

Raman spectroscopy may be used to study the chemical composition and construction of spectral images of various compounds, and has proven to be a useful tool for a variety of scientific fields. However, Raman scattering has intrinsically low cross-section, yielding low signals. Furthermore, conventional Raman spectroscopy with a multichannel array detector, such as the charged-couple device (CCD) camera, is limited by the inherent read noise of the detector electronics. These array-based spectrometers disperse different wavelengths of light onto separate detector pixels (or wavelength channels). Because of the inherent read-noise associated with CCD measurements, array-based Raman spectrometers have a major drawback in the low-signal regime. For example, if 100 photons are distributed over 100 pixels of a CCD detector, then the resulting signal in each pixel would be well below the typical CCD read-noise (of a few counts per channel), essentially rendering the Raman photons undetectable. As a result, Raman measurements often require collection times on the orders of hundreds of milliseconds or longer to obtain a spectrum with decent signal-to-noise ratio. This limitation hinders the use of Raman spectroscopy for hyperspectral imaging applications, where thousands to millions of different spatial points are measured. For example, the collection of a one-megapixel image would take 12 days with a typical 1 s per spectrum acquisition rate.

Recent advances in spatial light modulators (SLM) bring about a paradigm shift in the way that Raman imaging is performed. SLMs provide a means of producing variable programmable filter functions. Raman light is modulated by the filter functions loaded on SLMs, sent to the single-channel detector and recorded. The introduction of compressive data collection strategies enabled by SLMs allow fast Raman measurements by multiplexing Raman photons from different wavelengths onto a low-noise single channel detector [1,2,3,4,5,6,7,8,9,10]. Therefore, the scanning speed is basically only defined by the limits of the count rate of the detector. These recent studies have led to the conclusion that digital (binary) SLM optical filters created using the Digital Micromirror Device (DMD, Texas Instruments, Dallas, TX, USA ) can be as effective in data compression as analogue SLMs, with a simpler and more robust optical design [1,3,4,6,7,8,9,10]. Moreover, two detectors can be used to detect all the collected Raman photons, and thus increase compression speed, with appropriately optimized digital filters [7].

Hyperspectral Raman imaging is the combination of two technologies: spectroscopy and imaging, whereas compressive hyperspectral imaging combines three technologies: spectroscopy, imaging, and signal processing. In the hyperspectral imaging mode, an image is acquired by recording the full spectrum at each x, y point on the sample, which produces ‘data cubes’, where signal intensity is measured as a function of the x and y spatial dimensions and a spectral (wavenumber, cm^−1^) dimension. More generally, in hyperspectral imaging each pixel contains two-dimensional spatial information (x and y), and a third dimension of spectral information (e.g., wavenumbers). Subsequently, to derive the sought information from this large, multidimensional data set, in order to obtain a chemical image, it is necessary to transform it to lower-dimensional space through multivariate data analysis algorithms, such as principal component analysis (PCA), partial least squares (PLS), multivariate curve resolution (MCR), or total least-squares (TLS) [11,12], or in some cases through univariate analysis if a component of interest has a unique spectral peak (that has not overlapped with peaks due to other components) [13]. Generally, in a univariate analysis a property of interest is calculated based on a single value, which is correlated to the property to which that peak corresponds, such as the amount of that component at each image pixel location. In other words, the area (intensity) of the peak of interest is used as a measure of the amount of that component at each spatial location in the image. When an isolated peak of the investigated component does not exist, then more advanced multivariate data analysis techniques are typically used to extract the desired information. In fact, even when one or more components do have an isolated peak it can still be advantageous to perform a multivariate data analysis, as this will make use of the information contained in the entire measured spectrum.

Compressive Raman spectroscopy is similar to conventional hyperspectral Raman spectroscopy, except that the array detector is replaced by a spatial light modulator (SLM), and one or two single channel detectors, such as photon counting amplified photodiodes or photomultiplier tube detectors. In the compressive detection (CD) mode, instead of recording the full spectrum at each pixel, the spectral response (a score value) for each SLM filter function is recorded. In other words, CD differs from the conventional Raman detection in that the scores are directly detected using the hardware, rather than by obtaining scores after post-processing the full spectra. The total number of photons transmitted through each filter on the SLM is counted using a photon-counting detector, to obtain a direct measure of the score value. More specifically, CD effectively measures the dot-product of the filter vectors and the spectral vector coming from the sample. Chemometric techniques may be used to create optical filter functions, which are trained using full-spectral reference spectra. Once the filters are generated, photon counting is performed in each pixel (instead of full-spectral acquisition as in conventional systems). Because all the light transmitted by SLMs is measured by a single-channel detector rather than being separately detected by multiple channels (often >1000 channels), CD benefits from Felgett’s (or multiplex) signal-to-noise ratio (SNR) advantage. Thus, for example, a Raman spectrum with a total number of 100 photons would have a SNR of ~10 on a single-channel detector while, as previously noted, the same 100 photons would have been practically undetectable on a multi-channel CCD detector.

Here we review the latest advances in the design and performance of compressive hyperspectral Raman strategies, which facilitate the rapid collection of chemical images by directly applying programmable optical filters optimized to distinguish compounds of interest using spatial light modulators and single channel detectors.

## 2. Compressive Detection (CD) Strategies

Two design strategies were recently described for compressive Raman detection, in which filter functions are applied to either a digital micromirror array device (DMD) [1,3,14,15,16] or analog-based liquid crystal [2,6,17] spatial light modulators (LC-SLM). DMDs provide binary states, as each mirror pixel can be programmed to be either “on” or “off”, corresponding to a mirror tilt of ±12°. LC-SLMs, on the other hand, use light polarization to produce either phase or amplitude modulated variable analog filters [18]. Each pixel on LC-SLMs is a separately addressable optical phase modulator, which is used to rotate the polarization of the detected light between 0 (*p*-polarized) and 90 (s-polarized). The filter functions on liquid crystal cell control the degree to which, for example, the input *p*-polarized signal is rotated to s-polarization and thus reflected into the detection optical path. In this way, the spectral component that became s-polarized by means of the liquid crystal cell is entirely transmitted to the single channel detector while no *p*-polarized light reaches the detector. Earlier applications using transmissive LC-SLMs in compressive spectroscopy suffered from low light throughput of ~20% [6]. Recent developments in reflectance LC-SLM with higher light throughput (~80%) and fill factor [19] made possible for better performing spectrometers [2,20].

The data obtained in CD technology is fundamentally photon counts, which essentially corresponds to the dot product of filter functions and the spectra vectors. The filter functions are simply the combination of wavelengths specifically designed to regenerate the eigenvectors (often referred to principal component) obtained from chemometric algorithms. Multivariate techniques like PLS and PCA are appropriate to use to generate the optimal eigenvectors for a given experiment when the components are known [21,22]. These techniques use pure component samples as a built-in calibration set [23,24,25]. However, if components are not known then techniques like MCR may be more valuable to extract component information [26]. The amplitude of the measured signal is proportional to the amplitude of the eigenvectors, and thus to the amount of the corresponding compound.

Filter functions ensure that only the photons with certain wavelengths that are the most effective in discriminating the components of interest are detected. Irrelevant photons are disposed. To create filters for a given application, one must first obtain a high SNR training spectrum of each component of interest. Both LC-SLM and DMD-based CD systems can also function as a general Raman spectrometer to obtain full Raman spectra by notch scanning the SLM arrays one array column at a time. In DMD-based systems notch scanning could be performed by sequentially directing one mirror column towards the detector while all others are directed away, and count the number of photons at each notch position. In LC-SLM systems, SLM is used to produce band-pass filters with variable center wavelengths. However, the efficiency of band-pass scanning (or notch-scanning) is quite low since most Raman photons are discarded. An advantageous alternative to notch-scanning may be to use Hadamard [27] filter functions to obtain full spectra at higher SNR [2,17,23,24,28]. The efficiency of Hadamard strategy comes from that half of the Raman photons is always detected by each Hadamard filter. 

The present review is focused primarily on recent developments in DMD-based CD systems.

### Digital Micromirror Device (DMD)-Based Compressive Raman Detection

DMD is a micro-electronic mechanical system (MEMS) which consists of thousands of individually addressable moving micromirrors controlled by underlying electronics. DMD is also an SLM as the mirrors are highly reflective and are used to modulate light; to rotate the light to either a +12 degree or −12 degree position relative to the flat state of the array depending on the binary state of the cell below each pixel. These two positions determine the direction that light is deflected. Each tiltable mirror-pixel can be moved to reflect light to, or away from an intended target [29,30,31,32].

In DMD-based Raman detection systems the micromirrors on DMDs are horizontally binned (x mirrors/pixels) and vertically fully binned. That is, all mirrors in each column of the array are set to the same angle of either −12 or +12 and mirrors in each row are divided into adjacent groupings. Bins are defined by bands of photon energy, then groups of *x* adjacent columns are set in unison. The filter functions on DMD, then tells which columns of pixels are turned “on”, sending those selected photons to the detector and which columns are turned “off”, directing those photons away from the detector. More specifically, while photons with certain energy levels corresponding to “on” columns are collected in a single-channel detector and recorded, photons with wavelengths reaching “off” columns are disposed.

The first reported use of DMD SLMs in spectrometry dates back to 1995 by Wagner et al. [33]. In this early work the contrast ratio of the DMD was only about 60:1; today it goes as high as 2000:1 with a higher fill factor, allowing the design of better performing compressive Raman systems. Two approaches were reported recently to construct binary filters for DMD based-Raman systems [15,34]. The filter design developed by Scotte et al. is based on maximizing the precision of the components proportion estimates [15,35] using a new Cramer-Rao lower bound based algorithm. Buzzard and Lucier’s approach was to minimize the error in estimating photon emission rates of the chemical species investigated [34,36]. Both approaches based their theory on the fact that the photons transmitted through filter functions are modeled by Poisson random variables when the measurements are photon-noise limited [1,7,15,34,35,36,37]. Here, optimized binary compressive detection (OBCD) procedures based on Buzzard and Lucier’s approach is overviewed.

OBCD design: The recently-developed optimized binary compressive detection (OBCD) method relies on binary filters, which provides optimal measurement settings. Input data to be modeled to generate filter functions are photon counts, modeled by Poisson random variables whose variances equal to their means. Photon emission rates are correlated to the concentration of components of interest. In other words, concentrations are not directly measured, rather photon emission rates of each compound are estimated and the concentrations are calculated from this estimation. Objective is to minimize the mean square error between estimated and true emission rates. 

**Figure 1:**
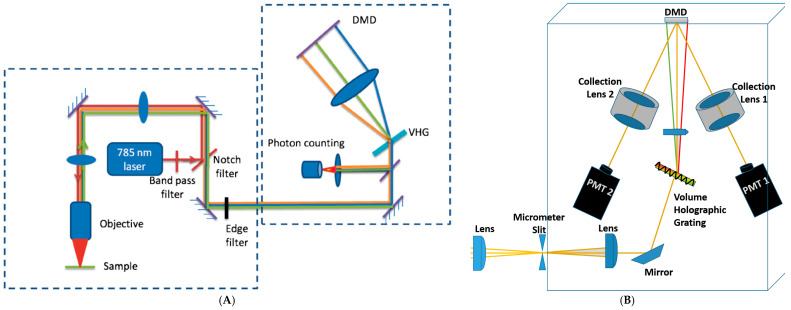
(**A**) Schematic of optimized binary compressive detection (OBCD) Raman system with 785 nm laser source (Reprinted from Reference [1] with permission from Elsevier); (**B**) schematic of Dual Detector CD (OBCD2) (detection module is shown only). Collimated beam of light is focused on a slit at the entrance of the spectrometer. The beam is recollimated and dispersed using a volume holographic grating (VHG). The dispersed beam is focused onto the DMD. The DMD can send parts of all the light towards either detector.

OBCD design has been shown to enable high-speed chemical classification, quantitation, imaging [1,36,37], as well as facilitating Raman classification in the presence of fluorescence background [8]. The design of an OBCD Raman spectrometer with 785 nm laser excitation whose schematic is shown in Figure 1A is described in detail in reference [1]. This design is configured to collect backscattered Raman photons with the same objective lens used to focus the excitation laser onto the sample. After separating Rayleigh photons using dichroic and notch filters, then Raman light is directed to the spectrometer module. It is then dispersed onto the DMD ((Texas Instruments, DLP D4000, 1920 × 1080 aluminum mirror array with 10.8 μm mirror pitch) after passing through volume holographic grating (VHG). In this design, 15 columns of adjacent mirrors are binned to yield a total of 128 bins, each bin corresponding to ~30 cm^−1^ and the whole spectral window being ~200–1700 cm^−1^. The Raman light transmitted by the “on” mirrors (corresponding to +12 degree tilt of mirrors) is then sent to the low-noise photon-counting avalanche photodiode (APD) module (dark count rate of ~200 photons/s and no read noise). The input binary optical filters tell which mirrors will point toward (assigned value of one) or point away (assigned value of zero) the detector. Authors have demonstrated that the OBCD with 785 nm excitation can be used to rapidly quantify binary and tertiary liquid mixtures with known components, and also to generate chemical images of mixed powders as well as generating filter functions using the MCR algorithm to facilitate high speed chemical imaging of samples for which pure components spectra are not available. [37]. They reported that with the OBCD strategy, a mixture of glucose and fructose is discriminated with as low as ~10 photons per pixel, corresponding to pixel dwell time of ~ 30 μs.

In order to demonstrate the accuracy of the OBCD detection mode, pairs of liquid mixtures with various degrees of spectral overlap were tested. Classification error was found to vary both with the degree of overlap and acquisition time. Low to moderately overlapping spectra (benzene/acetone with a correlation coefficient of 0.12, and n-hexane/methylcyclohexane with a correlation coefficient of 0.71) were accurately classified with as few as 10–25 photons per measurement in tens to hundreds of microseconds. The highly overlapped case of n-heptane/n-octane mixture with a correlation coefficient of 0.99, correct classification was achieved with ~200 photons in a few milliseconds. These acquisition times obtained using OBCD strategy were not accessible using comparative CCD-based Raman spectroscopy. 

Another OBCD Raman spectrometer prototype with 514 nm laser excitation with similar design to the 785 nm system mentioned above was also prototyped in Ben-Amotz’s lab [8]. For this design, a DMD chip of 608 × 684 mirror array with 10.8 μm mirror pitch was used. Two columns of adjacent mirrors were binned to give a total of 342 bins with each bin corresponding to 12 cm^−1^ and yielding a spectrometer with a ~200–4100 cm^−1^ spectral window. As a single channel detector, a photomultiplier tube (PMT) with a dark count rate of ~500 photons/s was used in this design. In this work [8] the feasibility of the OBCD strategy for Raman imaging of moderately fluorescing samples was demonstrated. A strategy for fitting a fluorescence background to the third-degree Bernstein polynomials was adopted to train OBCD filters, which were then used to quantitatively separate Raman signals from the fluorescence background, facilitating Raman imaging of chemicals in the presence of a fluorescence background.

OBCD2 design: In the OBCD detection strategy only a fraction of Raman photons, which were transmitted by “on” (+12 degrees) stage of micromirrors, were read by the detector. Raman light reaching to “off” (−12 degrees) micromirrors on DMD was disposed. A new strategy, termed as OBCD2, was proposed to increase the efficiency of Raman detection, wherein binary filters were generated in pairs [7]. Two detectors were used to count all Raman photons transmitted by two complimentary OB filters. OBCD2 is considered a derivative of OBCD, accordingly many of the assumption made in formulating the OBCD strategy [1,7,36] remain valid for OBCD2 strategy, as well.

A schematic of this technique is shown in Figure 1B. In the OBCD2 strategy, when one OBCD filter is generated corresponding to the “on” mirrors on DMD, the exact complement of that filter is also generated for implementation to “off” mirrors. To describe a system with *n* components a minimum of 2(*n* − 1) filters, which constitutes to *n −* 1 pairs of complementary filters, are required. Photons of different wavelengths are selectively reflected by micromirrors either positive 12 degrees or negative 12 degrees to the surface of the DMD and are directed to either one or the other PMT detector (dark count of ~500 photons/s) shown in Figure 1B. With OBCD2 filtering strategy all Raman photons are detected. As a result, Raman scattering rates recovered using OBCD2 filters have lower variance than those using OBCD filters [7]. In order to quantify the performance advantage of the OBCD2 over OBCD strategy, a ternary system of benzene, hexane, and methylcyclohexane were analyzed in [7]. For this system there were three OBCD filters and 2 × (3 − 1) = 4 OBCD2 filters (or two complementary pairs). The standard deviations of the estimated recovered Raman scattering rates are shown to improve ~63%, ~23%, and ~24% for benzene, hexane, and methylcyclohexane, respectively.

## 3. Assessment of DMD-Based Raman vs. CCD-Based Raman Detection

The performance of compressive Raman detection has been assessed compared to conventional Raman measurements by Scotte et al. in a recent paper [15], and by Ben-Amotz et al. in a forthcoming publication. Scotte et al. evaluated the performance of a custom-built DMD-based system with two commercially available spectrometers with different detectors (CCD and EMCCD) for the detection of low concentrations of biologically relevant components (microcalcification powders relevant to human breast cancer) [15]. They reported that in the high signal regime CD technology outperformed Raman imaging of the biological system they studied with a ×100 to ×10 speed improvements compared with CCD and EMCCD-based Raman imaging, respectively. In the low signal regime where noise is the limiting factor, they chose to compare the systems’ limits of detection (LOD). The LOD is defined as the minimum Raman light needed to reach on the detector to be able to correctly estimate component proportions. LOD for a compressive system is found to be similar to EMCCD and up to ×100 higher than CCD system. At equal signal-to-noise ratio CD is still faster than hyperspectral imaging. However, it is important to note that the custom-built DMD-Raman system in this work can further be improved as PMT used only has 40% quantum efficiency (QE) while two cameras used in commercial systems reach 90 to 95% QE.

**Figure 2:**
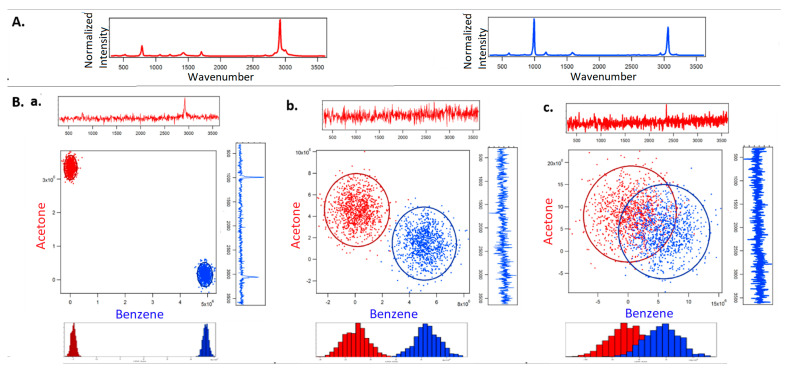
The figure shows the classification of acetone and benzene using a charge coupled device (CCD)-based Raman spectroscopy. Panel (**A**) shows the normalized spectra of acetone (red, top left) and benzene (blue, top right) collected using 30 mW laser power at the sample, and 1 s total spectral acquisition time to be used as training spectra in least square analysis. Subpanels (**a**–**c**) in Panel (**B**) correspond to the 1000 full spectral least squares classification of acetone and benzene in 1 ms using 30 mW, 3 mW, and 1 mW laser power at the sample, respectively.

**Figure 3:**
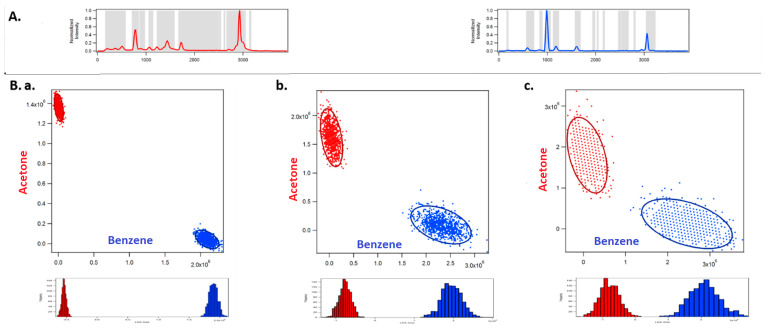
The figure shows the classification of acetone and benzene using optimized binary compressive detection (OBCD) filtering. Panel (**A**) shows the normalized spectra of acetone (red, top left) and benzene (blue, top right) collected to be used as training spectra to generate OBCD filters. This panel also shows OBCD filters in gray, i.e., the OB wavelengths that are multiplexed onto one PMT. Sub panels (**a**–**c**) in Panel (**B**) correspond to 1000 OBCD classification of acetone and benzene in 1 ms using 30 mW, 3 mW, and 1 mW laser power at the sample, respectively.

**Figure 4:**
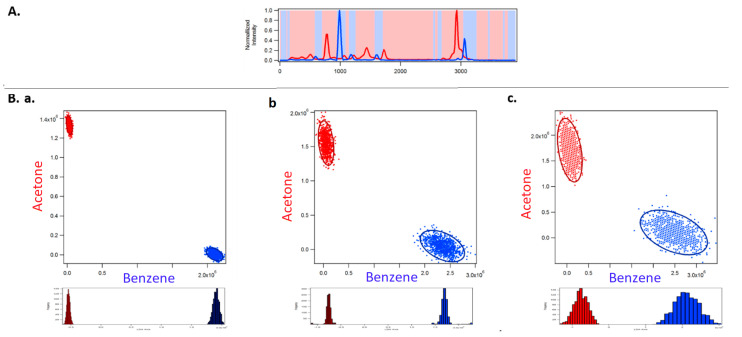
The figure shows the classification of acetone and benzene with OBCD2 filtering (using complementary OBCD filters). Panel (**A**) shows the normalized spectra of acetone (red, top left) and benzene (blue, top right) collected to be used as training spectra to generate OBCD2 filters. This panel also shows OBCD2 filters (shaded red and blue), i.e., the OB wavelengths that are multiplexed onto two PMTs. Sub panels (**a**–**c**) in Panel (**B**) correspond to 1000 OBCD classification of acetone and benzene in 1 ms using 30 mW, 3 mW, and 1 mW laser power at the sample, respectively.

Figure 2 shows the classification of acetone and benzene using full spectral acquisition using a CCD detector. Figure 3 corresponds to optimized binary compressive detection (OBCD) measurements, and Figure 4 shows the results for OBCD2 strategy performed under otherwise identical conditions to those used to obtain the results in Figure 2. In all three figures (Figure 2, Figure 3 and Figure 4), panel A shows training spectra obtained using 30 mW of laser power and an integration time of 1 s, while panel B shows classification results obtained using lower laser powers and integration times. More specifically, Panel A in Figure 2, Figure 3 and Figure 4 shows the normalized training spectra of acetone (red, top left) and benzene (blue, top right). Training spectra for the OBCD and OBCD2 measurements were obtained by measuring counts through Hadamard filters applied on the DMD for 1 s each (again with 30 mW of laser power). The counts were then invers Hadamard transformed to produce the high SNR spectra shown in Panels A of Figure 3 and Figure 4.

Panel B in Figure 2, Figure 3 and Figure 4 are each subdivided into three sub panels a, b, and c corresponding to the classification of acetone and benzene in 1 ms using 30 mW, 3 mW, and 1 mW of laser power at the sample, respectively. The normalized spectrum of acetone (red) and benzene (blue) at each laser power are shown on the top and the right of the two-dimensional classification plot in each sub panel. The ellipses represent the 95% confidence interval.

Note that the CCD cannot collect spectra faster than 1 ms, which is why the laser power is chosen to be reduced rather than reducing integration. However, the CD measurements can be performed using the PMT detectors with integration times as short as ~3μs—this is a key advantage of CD-based (OBCD or OBCD2) measurements as opposed to conventional full spectral CCD-based measurements.

In the assessment of CCD based-Raman in Panel B of Figure 2, each cloud consists 1000 independently measured spectra classified by post processing the spectra using least squares to extract the lower dimension concentration information. The bottom of panel B shows the dimension reduced linear discriminant analysis (LDA) histograms, which make it visually clear that the classification error increases with the reduction in signal (Raman scattered photons). Note Panel B of Figure 2 also includes the full spectra obtained in 1 ms of integration using the three laser powers. These spectra clearly reveal that no spectral peaks are evident at laser powers of 3 mW and below. However, the LDA histogram reveal that it is still possible to accurately discriminate the acetone and benzene spectra at a power of 3 mW using such extremely noisy spectra. 

Figure 3 shows the results obtained using OBCD filtering strategies for the same classification problem. OBCD filters are shaded in gray in panel A of Figure 3. These shaded wavelengths are the OB wavelengths, that were applied programmatically onto a DMD, then multiplexed onto one PMT. Each cloud consists of 1000 independently measured intensities (photon counts) obtained using two sequentially applied filters onto one PMT detector. OBCD2 filters are shaded in red and blue in panel A of Figure 4 All Raman photons reflected by the DMD are detected using two PMTs in OBCD2 strategy. The photons from both PMTs are used to transform counts to concentration space and are shown in the classification plots in panel B of Figure 4 Panel B shows OBCD2 classification plots and LDA histograms of acetone and benzene. Comparison of these LDA histogram results with those in Figure 2 clearly reveals the greater discriminating power of CD-based as opposed to CCD-based measurements, as well as the fact that OBCD2 outperforms OBCD.

Table 1 compares the resolution of LDA histograms of acetone and benzene produced by different classification strategies and using different laser powers. The resolution (R) was calculated using Equation (1) below. It is defined by the ratio of the absolute separation in the mean values (μ) of the clouds obtained from the acetone and benzene histograms divided by the sum of the standard deviation (σ) of each of the histograms. The higher the resolution, the greater the separation between the histograms and the better the classification between the two chemicals.

(1) $$R=\frac{\left|\mu_{1}-\mu_{2}\right|}{\sigma_{1}+\sigma_{2}}$$

The best resolution is achieved for the highest laser power at the sample. This makes sense because higher laser power generates more Raman photons yielding a higher signal-to-noise. The resolution for the three classification methods are comparable at the higher laser power. However, for lower laser powers, the resolution of classification of acetone and benzene is greatest for the OBCD2 classification, followed by OBCD and CCD classifications. 

**Figure 5:**
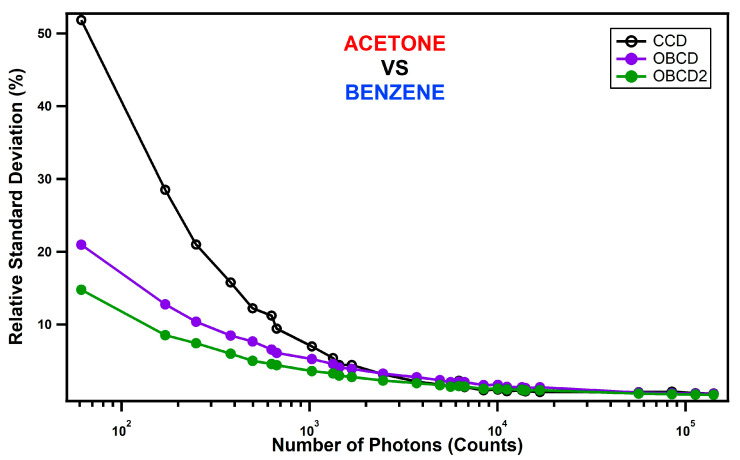
The figure compares the relative standard deviation in the classification using CCD, OBCD and OBCD2. The instrument was set to laser powers 30, 3, and 1 mW and counts were measured at nine integration times; 100, 80, 60,40, 10, 8, 6, 4, and 1 ms at each power.

Figure 5 compares the relative standard deviations (RSD, Equation (2)) in the classification using CCD and optimal binary CD measurements. The higher the RSD the worse the classification. The *x*-axis in Figure 5 represents the total photon counts, which were measured by adjusting both the laser power and the integration times. The same Raman photons were sent to the CD spectrometer as the CCD spectrometer by using a flip mirror to direct the light either towards the CCD or OBCD detection systems.

(2) $$RSD=\frac{\sigma }{\mu }\times 100$$

In the high intensity (photon count) limit, the *RSD* for all three methods were comparable. However, below about 1000 photons the OBCD classification has lower *RSD*. Below about 2000 photons the OBCD2 classification has lower *RSD*. Thus, although full hyperspectral CCD measurements are always a bit better than OBCD and OBCD2 measurements in the high intensity limit, in the low signal regime both OBCD and OBCD2 far outperform CCD-based measurements, and OBCD2 is slightly better than OBCD. Additional measurements (not shown) performed using components that are more highly overlapped Raman spectra have been found to have similar crossover points. Thus, it appears to be generally true that OBCD/OBCD2 measurements outperform conventional full spectral CCD-based measurements when the integrated number of photoelectrons in the spectra is below a few thousand counts.

## 4. Discussion

A key bottleneck to fast Raman analysis, including real-time monitoring and hyperspectral imaging, is the time required to acquire multivariate hyperspectral data and post-processing the full spectra. Multichannel detectors (e.g., CCD) are generally more expensive and less sensitive than single channel detectors and also require cooling when long integration time and low dark counts are needed. A CCD-based Raman spectrometer cannot operate fast enough to be applicable for dynamic system measurements. A compressive spectrometer, which incorporates SLM technology and a single channel detector, offers not only higher sensitivity and speed, but also a potentially lower-cost alternative to CCD-based Raman imaging. Most importantly, compressive detection can be used to obtain chemical imaging information in the very low signal limit at which conventional CCD-based Raman spectroscopy is completely impossible. 

Here we overviewed the latest advances in compressive Raman detection with focus on the optimized binary compressive detection (OBCD) strategy. At the heart of OBCD strategy, is the widely adopted reflective light modulator DMD used in standard computer projection systems (manufactured by Texas Instruments), whose switch speed, contrast ratio, and broad spectral capability outperforms analog-based SLMs. DMD is a semiconductor-based “light switch” array of hundreds of thousands of individually switchable mirror pixels. The light switching speeds in the order of kHz at which each mirror can modulate between “on” and “off” states enable CD measurements at kHz frequencies. DMDs have faster modulation rates [38]. Analog-based SLMs, on the other hand, do not have the speed and precision capability which make the DMD more attractive for use in Raman spectroscopy. Also, they have slower pixel response and have to operate on linearly polarized light. DMDs maintain higher throughput due to polarization insensitivity.

Rapid evaluation of chemical species in complex chemical matrices is of high importance in a diverse array of applications. The projected advances brought about by DMD use in Raman spectroscopy will make real-time measurement possible, for example real-time imaging systems for medical and scientific communities, in-line quality inspections to evaluate chemical compositions, etc. DMD-based Raman systems are shown to effectively suppress laser-induced fluorescence backgrounds, which makes fast Raman mapping of samples with large background possible [8,9]. In a very recent publication by Scotte et al., the performance of DMD-based compressive Raman technology is assessed on a biologically relevant sample mimicking microcalcification in breast cancer [15]. For this study, four micromirrors on DMD were binned horizontally, giving a spectrometer with spectral resolution of 40 cm^−1^ and the spatial resolution of 1.4 μm with 532 nm laser excitation. At these parameters, compressive Raman spectroscopy is reported to be a very useful technique correlating the state of the cancer to the chemical composition of microcalcification.

In addition, the studies in references [23,24] showed the potential of compressive Raman detection as a process analytical technology (PAT) tool for pharma industry. Non-invasive, real-time measurement systems for qualitative and quantitative analysis of raw, in-process, and finished products in continuous pharmaceutical manufacturing settings are quite critical in the success of PAT program initiated by US FDA in 2004 [39]. CD-Raman spectroscopy speeds up the collection of Raman data, which makes it attractive as a PAT tool for real-time measurement applications in continuous manufacturing.

CD-Raman systems can reproduce the functionality of conventional array-based Raman spectroscopy to collect full spectral information by raster-scanning each array column. However, it is important to emphasize that the full speed advantage is only realized when it is used in a compressive detection mode with filter functions. Furthermore, compressive Raman detection is most advantageous when it is used in low signal regime or high-speed conditions. A CCD cannot acquire at high speeds, but a single channel detector such as PMT can. Full spectral measurements may be preferred under high-signal conditions where dark and read-noise do not affect SNR of spectra or in cases where the number of filter functions may be too many to be practical. With CCD measurements, there is no loss of data due to information compression and full spectral data can be further investigated in the future. However, in low photon budget OBCD is advantageous because multiplexing on a single channel detector increases the SNR dramatically. 

## Figures and Tables

**Table 1:** The table compares the resolution of the linear discriminant analysis (LDA) histograms of acetone and benzene produced by different classification strategies and using different laser powers.

Classification Strategy	30 mW, 1 ms	3 mW, 1 ms	1 mW, 1 ms
CCD	26	3	1
OBCD	22	7	4
OBCD2	32	13	6
